# Knowledge-Guided Modulation for Terrain-Aware Landslide Detection Using Deformable Transformers

**DOI:** 10.3390/s26092813

**Published:** 2026-04-30

**Authors:** Yan-Chang Jia, Shu-Yan Hua, Hong-Fei Wang, Tong Jiang, Qi-Qi Zhao

**Affiliations:** 1 College of Geosciences and Engineering, North China University of Water Resources and Electric Power, Zhengzhou 450046, China; jiayanchang@ncwu.edu.cn (Y.-C.J.); jiangtong@ncwu.edu.cn (T.J.); z20241110969@stu.ncwu.edu.cn (Q.-Q.Z.); 2 Yellow River Engineering Consulting Co., Ltd., Zhengzhou 450003, China; wanghf_yrec@163.com

**Keywords:** landslide detection, remote sensing, deformable DETR, knowledge-guided learning, multi-channel data fusion, terrain information, transformer-based object detection

## Abstract

**Highlights:**

**What are the main findings?**

- A knowledge-guided five-channel Deformable DETR is proposed to jointly exploit optical and terrain-derived cues (e.g., DEM and slope) for robust landslide detection in complex mountainous scenes.
- Injecting terrain-aware priors into multi-scale deformable attention improves localization precision and convergence stability beyond conventional multi-channel fusion baselines.

**What are the implications of the main findings?**

- The framework integrates data-driven transformers with terrain-related priors, improving terrain-aware representation learning for landslide detection in remote sensing imagery.
- The proposed design shows potential for extension to other terrain-controlled hazards and may support scalable inventory mapping in geomorphologically complex regions.

**Abstract:**

Landslide detection using medium-resolution optical remote sensing imagery remains challenging in complex mountainous environments because of spectral ambiguity, vegetation cover, shadows, and background interference. Although recent deep learning methods have improved detection performance, most existing approaches remain primarily appearance-driven and do not explicitly exploit terrain-related priors that are closely associated with slope instability. To address this limitation, we propose a terrain-aware deformable transformer framework for landslide detection using multimodal remote sensing data, in which RGB imagery, DEM, and slope are jointly incorporated through a unified five-channel representation, and a knowledge-guided modulation module is introduced to enhance feature learning using terrain priors derived from DEM and slope. Here, “knowledge-guided” refers specifically to explicit topographic priors rather than complete geological or hydrological knowledge. Experimental results on the Bijie landslide dataset show that the proposed method outperforms several competitive baselines and achieves 72.9% AP@[0.5:0.95] and 77.2% AP75, while improving localization robustness in visually confusing mountainous scenes. These results indicate that terrain-aware feature modulation can improve geomorphological plausibility and detection accuracy for landslide inventory mapping, although further cross-region validation is still needed to assess broader generalization.

## 1. Introduction

Landslides are widespread natural hazards that pose significant threats to human life, infrastructure, and regional stability [1,2,3,4]. Rapid and accurate landslide inventory mapping is essential for post-event response, hazard assessment, and risk mitigation, particularly in complex mountainous regions [5,6,7,8].

Remote sensing has become an indispensable tool for landslide investigation because it provides synoptic, repeatable, and large-scale observations over inaccessible terrain [9,10,11,12,13]. Optical imagery, aerial photographs, and digital elevation models (DEMs) have been widely used for landslide detection, inventory generation, and susceptibility assessment [5,8,9,10,11,12,13]. Early studies mainly relied on visual interpretation, object-based image analysis, and semi-automatic mapping techniques [11,12,13,14]. Bitemporal inventory mapping, multiscale segmentation, and statistically based susceptibility modeling have also provided useful methodological background for landslide mapping and hazard assessment [15,16]. More recently, machine learning and deep learning methods have been increasingly adopted to improve landslide detection efficiency and mapping accuracy, including CNN-based recognition, change-detection frameworks, semantic segmentation, and end-to-end inventory mapping approaches [17,18,19,20,21]. In addition to optical remote sensing and multi-source data fusion, interferometric synthetic aperture radar (InSAR) has become an important tool for landslide investigation, particularly for deformation monitoring, active landslide identification, and early instability analysis [22,23,24]. Compared with optical imagery, InSAR is more sensitive to subtle ground displacement and therefore provides complementary evidence for slope activity [22,23]. However, its effectiveness may be constrained by temporal decorrelation, geometric distortion, vegetation cover, and data availability, especially in steep mountainous environments [22,23]. For this reason, optical imagery combined with terrain-related auxiliary information remains a practical solution for large-area landslide inventory mapping, whereas InSAR-based observations are particularly valuable for dynamic monitoring and future multi-source integration.

Despite these advances, landslide detection using medium-resolution optical imagery (e.g., Sentinel-2) remains challenging in complex mountainous environments. Landslides often exhibit heterogeneous spectral characteristics and are frequently obscured by shadows, vegetation, and complex backgrounds, which hinder reliable target discrimination [9,11,12,13,17,18,19,20]. As a result, conventional RGB-based detectors are prone to producing false positives in spectrally similar but geomorphologically inconsistent regions.

Terrain-related learning and slope-stability studies further indicate that elevation, slope, and other topographic variables provide important physical context for landslide hazard assessment [25,26]. Recent advances in deep learning have substantially improved feature representation for remote sensing applications [27,28,29,30,31]. Convolutional neural networks and transformer-based architectures have shown strong capabilities in modeling spatial features and long-range dependencies [27,28,29,30,31]. Recent reviews and task-specific studies on remote sensing object detection have further emphasized challenges such as complex backgrounds, small objects, adaptive contextual modeling, and multi-scale feature representation [32,33,34,35]. However, most existing landslide detection methods remain largely appearance-driven and rely heavily on data-driven representations [17,18,19,20], without explicitly incorporating terrain-related information that fundamentally affects landslide occurrence.

From a geomorphological perspective, landslide occurrence is influenced by multiple factors, including terrain configuration, lithology, geological structure, hydrological conditions, land cover, and external triggers such as rainfall and human disturbance. Among these factors, DEM-derived variables such as elevation and slope provide accessible topographic proxies that are particularly useful for terrain-aware remote sensing analysis. Accordingly, DEM-derived variables have been widely used in landslide susceptibility modeling and mapping studies [5,10,16]. However, in many deep-learning-based detection frameworks, terrain information is introduced through simple early-fusion strategies, such as concatenating DEM-derived layers with RGB imagery [17,18,19,20,21,24]. These approaches do not explicitly enforce consistency between learned features and underlying terrain structure, and may therefore produce detections that are spectrally plausible but geomorphologically inconsistent.

To address these limitations, we propose a terrain-aware deformable transformer framework for landslide detection using multimodal remote sensing data. The proposed approach integrates spectral and topographic information through a unified representation and introduces a modulation mechanism that injects DEM- and slope-derived priors into multi-scale feature learning. Rather than representing complete geological knowledge, the introduced prior provides explicit topographic guidance for improving terrain-aware representation learning and reducing geomorphologically implausible detections in complex mountainous scenes.

The main contributions of this study are summarized as follows:

(1) We develop a terrain-aware landslide detection framework that jointly utilizes RGB imagery, DEM, and slope information for object-level landslide detection in complex mountainous regions;

(2) We introduce a lightweight modulation mechanism that injects DEM- and slope-derived terrain priors into multi-scale feature learning, going beyond simple early fusion of multimodal inputs;

(3) We demonstrate through comparative experiments that terrain-aware modulation improves detection accuracy and localization robustness on a region-level split of the Bijie dataset, while also discussing the limitations of DEM/slope-only priors and the need for broader cross-region validation.

## 2. Materials and Methods

### 2.1. Study Area and Dataset Description

The study area is located in Bijie City, Guizhou Province, southwestern China, a mountainous region characterized by strong topographic relief, dense vegetation cover, and frequent slope hazards. The area is generally dominated by middle- to low-mountain landforms, with complex terrain dissection and pronounced elevation differences. Under the combined influence of subtropical monsoonal climate, seasonal rainfall concentration, and local human disturbance such as road construction and slope cutting, landslides are widely developed in this region. In geological terms, the area contains heterogeneous lithological conditions and weathered slope-forming materials, which contribute to spatially variable slope stability. Therefore, Bijie provides a representative setting for evaluating terrain-aware landslide detection methods in geomorphologically complex environments.

The Bijie landslide dataset used in this study consists of optical remote sensing imagery and corresponding terrain-derived auxiliary data. Each sample includes an RGB image together with digital elevation model (DEM) and slope information, forming a five-channel multimodal input. The dataset covers diverse landslide scenarios, including vegetation-covered slopes, road-adjacent landslides, large irregular landslides, dark-background targets, and visually confusing disturbed surfaces. These characteristics make the dataset suitable for evaluating both appearance-based and terrain-aware landslide detection models.

**Figure 1:**
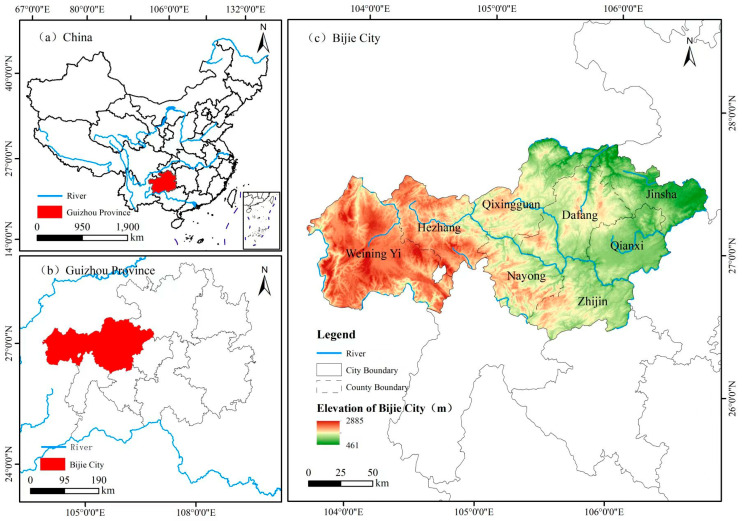
Location of the study area and topographic setting in Bijie City, Guizhou Province, China.

In addition to the topographic setting shown in Figure 1, the study area is characterized by complex geomorphic conditions and strong slope heterogeneity, which are important background factors for landslide occurrence. Since the present study focuses on terrain-aware detection rather than process-based susceptibility modeling, geological information is used here to provide regional context rather than as an explicit model input.

In this study, “satellite-scale (10 m)” refers to the relatively fine spatial detail of the adopted public dataset for operational inventory mapping; the optical imagery used is at 10 m resolution (Sentinel-2 MSI), and terrain layers are resampled to the same grid.

We utilized the landslide inventory (vector boundaries) from the Bijie dataset as the ground truth. Based on these locations, we independently acquired and pre-processed the corresponding Sentinel-2 (10 m) and ALOS PALSAR DEM (12.5 m) tiles via the Google Earth Engine (GEE) platform. The multimodal data sources and preprocessing steps are summarized in Table 1.

A quantitative summary of the dataset composition is further provided in Table 2, including the number of image tiles, landslide instances, and the object-size distribution of the available annotated subsets.

Small, medium, and large objects were categorized according to the COCO criterion based on bounding-box area.

The landslide inventory used in this study was derived from the publicly available Bijie dataset, and object-level annotations were generated in the form of bounding boxes based on landslide boundaries. To reduce spatial leakage, the dataset was partitioned at the regional level into training, validation, and test subsets, so that each geographic region appeared in only one split. Table 2 reports the quantitative statistics of the training, validation, and test subsets, including the number of image tiles, landslide instances, and the object-size distribution.

**Figure 2:**
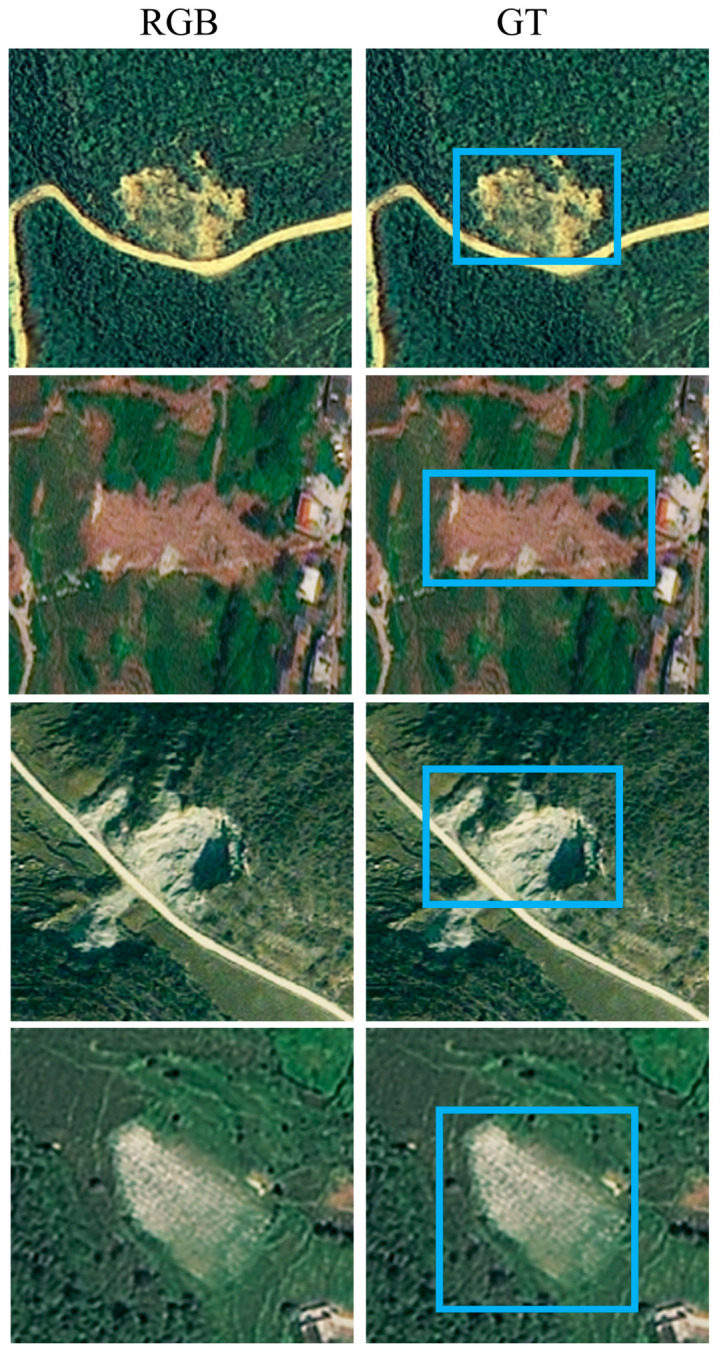
Representative training samples from the Bijie dataset. The examples illustrate the diversity of landslide appearance and background complexity in the training set, including road-adjacent, elongated, bare-soil-like, and weak-contrast targets.

Representative training samples are shown in Figure 2 to illustrate the diversity of landslide appearance and background complexity in the Bijie dataset.

All data layers were spatially aligned and resampled to a unified spatial resolution before model development. Detailed split settings and evaluation protocols are described in Section 3.2 Data Split and Evaluation Protocol.

### 2.2. Multimodal Input Construction

To effectively capture both spectral and terrain-related characteristics, a five-channel multimodal input representation is constructed, consisting of RGB imagery, DEM, and slope layers. This design enables the model to jointly learn appearance-based and terrain-constrained features, which are essential for robust landslide detection in complex environments.

The multimodal input is formulated as:

(1) $$X=Concat\left(X_{rgb},X_{dem},X_{slope}\right)$$

where *X_rgb_*, *X_dem_*, and *X_slope_* denote the RGB image, digital elevation model, and slope, respectively.

To ensure numerical stability and compatibility across modalities, the DEM and slope layers were normalized independently before stacking. All five channels were then fed into the backbone simultaneously, enabling joint feature learning from both spectral and topographic cues.

### 2.3. Baseline Deformable Transformer Detector

As the baseline detector, the standard Deformable DETR framework is adopted, which operates on RGB imagery and is capable of modeling long-range dependencies and multi-scale contextual information. Based on this baseline, we extend the input representation by incorporating DEM and slope information, forming a five-channel multimodal input for subsequent model variants.

The backbone network extracts multi-scale feature maps at different spatial resolutions, denoted as {*F*_1_, *F*_2_, *F*_3_, *F*_4_}, corresponding to increasing receptive fields. These feature maps are subsequently projected into a unified embedding space and passed to a multi-scale deformable transformer encoder–decoder. Object queries are used to iteratively attend to salient regions and generate final bounding box predictions through the detection head.

This baseline provides strong detection capability while serving as a fair comparison framework for evaluating the effectiveness of the proposed knowledge-guided modulation strategy.

### 2.4. Knowledge-Guided Modulation Module

In this study, “knowledge-guided” refers specifically to explicit terrain-related priors derived from DEM and slope, rather than complete geological, hydrological, or stratigraphic knowledge. To incorporate such priors into feature learning without introducing additional sensor modalities, we designed a lightweight modulation module. Unlike conventional fusion methods that rely only on direct channel concatenation, the proposed module uses a terrain-prior branch to dynamically reweight multi-scale feature responses before transformer encoding.

The knowledge-guided branch extracts terrain priors from DEM and slope layers to modulate multi-scale features. The layer-wise configuration of this modulation module is provided in Table 3.

First, the DEM and slope layers are encoded into a compact terrain-prior tensor, *P*:

(2) $$P=f_{\theta }\left(Norm(X_{dem},X_{slope})\right)$$

where *Norm* (·) denotes per-scene min–max normalization and $f_{\theta }$ is a shallow embedding network consisting of two 3 × 3 convolutions with ReLU activation, yielding a *d*_mod *el*_-channel prior feature.

For each backbone feature level *l*, the tensor *P* is resampled to the corresponding spatial resolution to generate a modulation mask, *M_l_*:

(3) $$M_{l}=\sigma \left(f_{\phi }(R\mathrm{es}ize(P))\right)$$

where $f_{\theta }$ represents a learnable 1 × 1 projection and σ(·) is the sigmoid function. The final modulated feature,$X_{l}^{\prime }$, is formulated as:

(4) $$X_{l}^{\prime }=(1+\alpha M_{l})⊙X_{l}$$

where ⊙ denotes element-wise multiplication, and we set *α* = 1.0 in all experiments. The KG modulation is applied to multi-scale features before deformable attention encoding.

It should be emphasized that the knowledge-guided signal is derived solely from the existing DEM and slope inputs and is injected internally through feature modulation. Therefore, for the comparison between M3 and M4, both models operate on the same five-channel input, and performance differences are attributable to the integration mechanism rather than additional input information.

### 2.5. Overall KG-5C-DETR Framework

The proposed KG-5C-DETR framework combines the five-channel Deformable DETR baseline with the knowledge-guided modulation strategy described above. After modulation, the multi-scale features are encoded through deformable attention to aggregate contextual information across scales, and the decoder predicts landslide bounding boxes and confidence scores in a single forward pass.

**Figure 3:**
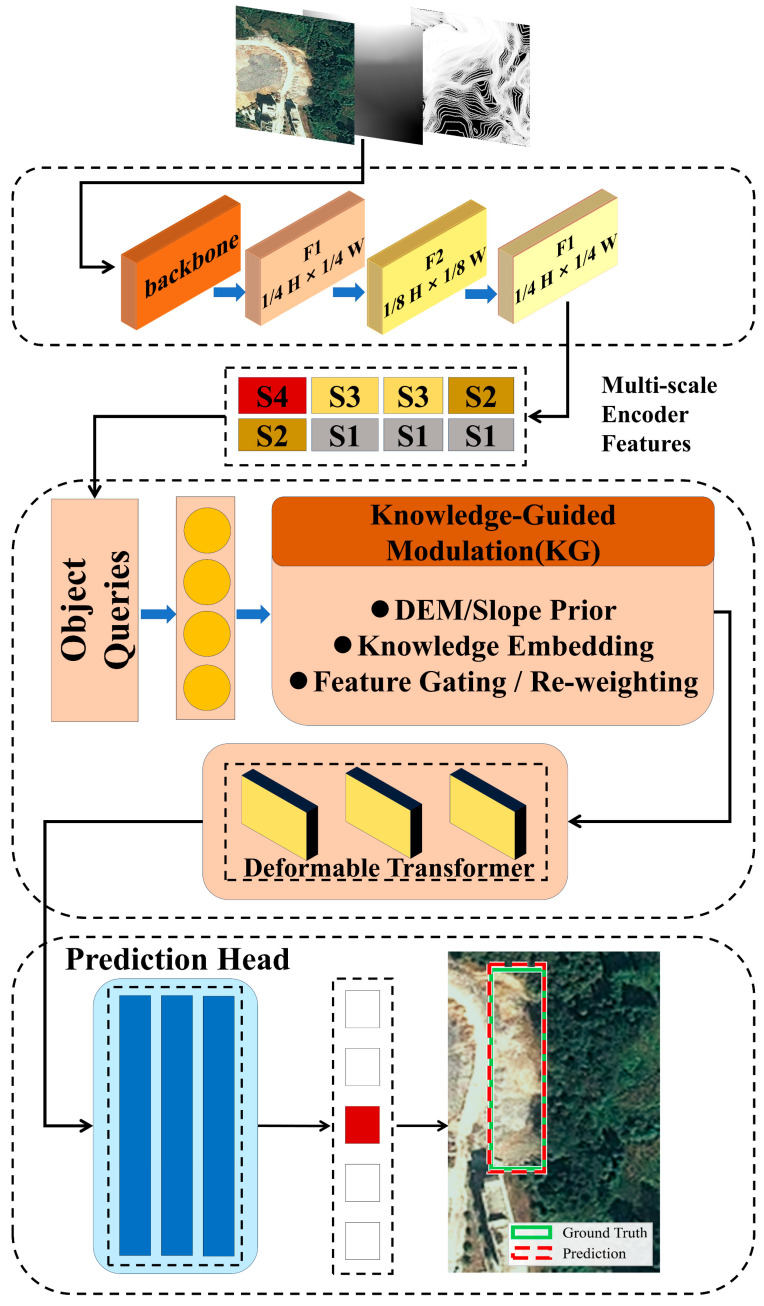
Overall architecture of the proposed knowledge-guided 5-channel Deformable DETR framework.

An overview of the complete framework is shown in Figure 3. The model preserves the efficiency and end-to-end detection characteristics of Deformable DETR, while enhancing its sensitivity to terrain-consistent landslide patterns through topographic prior modulation.

### 2.6. Loss Function

The models were trained end-to-end using a standard DETR-style objective combining classification and box regression:

(5) $$L=L_{cls}+\lambda_{1}L_{bbox}+\lambda_{2}L_{giou}$$

where *L_cls_* is the classification loss, *L_bbox_* is the L1 loss for bounding-box regression, and *L_giou_* is the generalized IoU loss. Following common DETR/Deformable DETR practice, we set $\lambda_{1}=5$ and $\lambda_{2}=2$ for all experiments.

## 3. Experimental Design

### 3.1. Comparative Model Settings

To evaluate the contribution of terrain information and the effectiveness of the proposed knowledge-guided modulation strategy, four comparative models were designed. M1 uses RGB imagery only and serves as the appearance-based baseline. M2 introduces DEM information to evaluate the contribution of elevation cues. M3 further incorporates slope information to assess the benefit of local terrain-gradient descriptors. M4, namely KG-5C-DETR, uses the same five-channel input as M3 but enables the knowledge-guided modulation module.

This comparison design makes it possible to distinguish the performance gain brought by multimodal input enrichment from that brought by the proposed integration mechanism. The detailed configurations of all compared models are summarized in Table 4.

### 3.2. Data Split and Evaluation Protocol

To assess model generalization under realistic deployment conditions, the dataset was partitioned at the regional level into training, validation, and test subsets. Each geographic region appeared in only one split, thereby avoiding spatial leakage between training and evaluation data.

Unless otherwise stated, all quantitative comparisons were conducted on the held-out region-level test set. The validation set was used for model selection, convergence monitoring, and hyperparameter tuning, while the test set was used exclusively for final performance reporting.

### 3.3. Training and Implementation Details

All models were trained under identical optimization settings to ensure a fair comparison. We used AdamW with a step learning-rate schedule. The initial learning rate was 2 × 10^−5^, with a backbone learning rate of 5 × 10^−6^, and the learning rate was decayed by a factor of 0.1 at epoch 150. Standard data augmentation included random horizontal flipping and multi-scale resizing with aspect-ratio preservation.

Implementation was based on PyTorch v2.4.0+cu121, and all experiments were conducted on a single NVIDIA RTX 4070 Ti GPU with 16 GB memory. The backbone was a ResNet-50 pretrained on ImageNet. The transformer configuration included 6 encoder layers, 6 decoder layers, a hidden dimension of 256, 4 feature levels, 8 attention heads, an FFN dimension of 1024, a dropout rate of 0.1, and 4 sampling points for deformable attention. The number of object queries was set to 300. Training was performed for 200 epochs with a batch size of 2. The complete implementation settings are summarized in Table 5.

### 3.4. Evaluation Metrics

Detection performance was evaluated using Precision, Recall, F1-score, and average precision (AP) under different IoU thresholds. In particular, AP50, AP75, and AP@[0.5:0.95] were reported to assess both detection accuracy and localization quality. Following the COCO-style evaluation protocol, AP@[0.5:0.95] was computed by averaging AP across IoU thresholds from 0.50 to 0.95 with a step size of 0.05.

In addition, Precision–Recall curves were used to analyze the trade-off between detection completeness and false positives across confidence thresholds.

## 4. Results

Unless otherwise stated, all quantitative results are reported on the held-out region-level test set (spatially separated by region).

### 4.1. Overall Quantitative Performance on the Held-Out Region-Level Test Set

Table 6 reports the test-set performance of the four model variants under identical experimental settings. Introducing DEM improves AP@[0.5:0.95] from 67.8% in M1 (RGB-only) to 69.5% in M2 (RGB + DEM), and further adding slope increases it to 71.5% in M3 (RGB + DEM + Slope). After enabling the knowledge-guided modulation module, M4 (KG-5C-DETR) achieves the best overall performance, reaching 72.9% AP@[0.5:0.95] and 77.2% AP75, with only a marginal increase in parameter count.

To provide a stronger comparison with widely used one-stage detectors, we additionally evaluated RGB-only YOLO baselines. Depending on the specific training configuration, the best AP@[0.5:0.95] values ranged from 50.9% to 63.4%, which remained lower than the RGB-only Deformable DETR baseline (M1, 67.8%).

Compared with M3, which uses the same five-channel input but relies on conventional early fusion, M4 improves AP@[0.5:0.95] by 1.4 points and AP75 by 1.0 point. Compared with the RGB-only baseline (M1), the gain reaches 5.1 points in AP@[0.5:0.95]. These results indicate that both terrain modalities and the proposed knowledge-guided integration contribute to the final performance improvement.

Notably, these gains are obtained with only a negligible increase in model size.

### 4.2. Training Dynamics and Convergence Behavior

**Figure 4:**
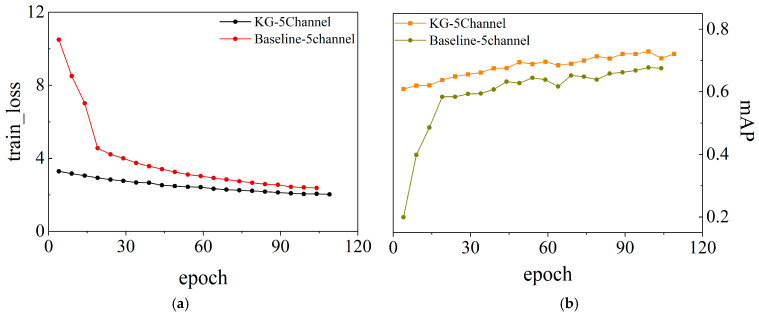
Training convergence comparison between Baseline-5Channel and KG-5Channel models. (**a**) Validation performance curves during training; (**b**) Training loss curves showing convergence behavior.

Training curves in Figure 4 are computed on the validation set, while final quantitative results are reported on the held-out test set (Table 6). Figure 4a shows the performance trajectories during training. Although both models improve steadily, KG-5C-DETR remains consistently higher, with a clearer advantage in the later training stage.

The training loss curves in Figure 4b further show that KG-5C-DETR converges faster and maintains a lower loss throughout most of the training process, indicating more stable optimization behavior.

### 4.3. Precision–Recall Characteristics and Robustness Under Stricter Overlap Requirements

**Figure 5:**
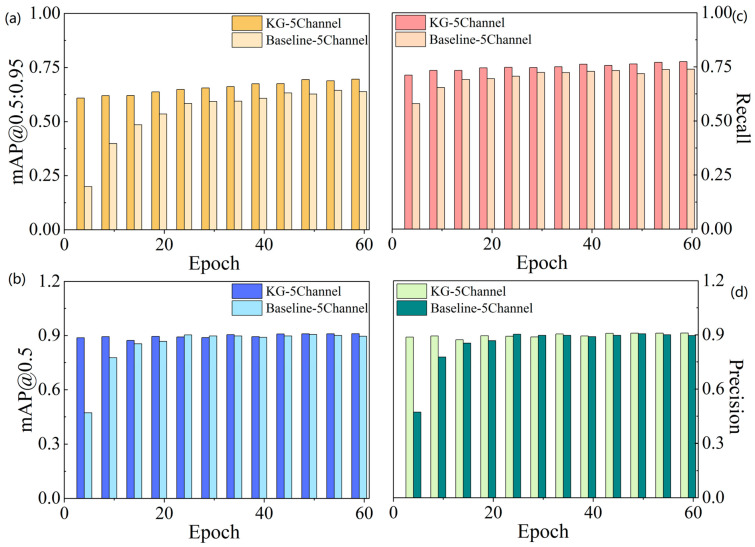
Evolution of key detection metrics during training. (**a**) mAP@[0.5:0.95]; (**b**) mAP@0.5; (**c**) Recall; (**d**) Precision.

To evaluate detection reliability during training, Figure 5a–d show the evolution of four key metrics, including mAP@0.5, mAP@0.5:0.95, precision, and recall, respectively. Across all four metrics, KG-5C-DETR consistently outperforms the five-channel baseline, indicating improved detection accuracy, localization quality, and false-positive control.

**Figure 6:**
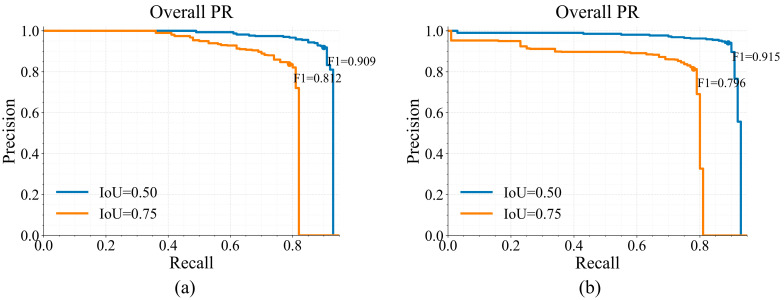
Precision–recall (PR) curves under different IoU thresholds. (**a**) KG-5C-DETR; (**b**) five-channel baseline.

Figure 6a and Figure 6b show the precision–recall (PR) curves of KG-5C-DETR and the five-channel baseline, respectively, with PR behavior reported at IoU = 0.50 and IoU = 0.75 in each panel. At both IoU thresholds, KG-5C-DETR maintains a more favorable precision–recall trade-off than the baseline. The advantage is particularly evident under the stricter IoU = 0.75 criterion, where the baseline exhibits a sharper precision drop at higher recall, whereas KG-5C-DETR preserves higher precision, indicating better localization robustness under stricter overlap requirements.

### 4.4. Qualitative Detection Results Under Complex Scenarios

**Figure 7:**
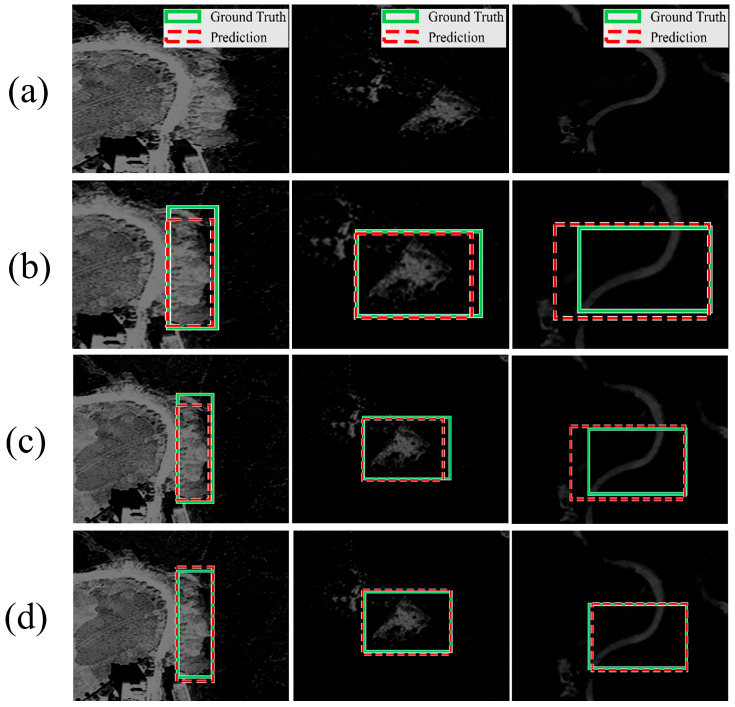
Qualitative comparison of detection results under low-contrast/dark-background conditions. (**a**) RGB image; (**b**) 3-channel DETR baseline; (**c**) 5-channel DETR baseline; (**d**) KG-5C-DETR.

Additional low-contrast and dark-background examples are presented in Figure 7. In Figure 7a–d, each column shows one representative case, while the four rows correspond to the RGB image, the 3-channel DETR baseline, the 5-channel DETR baseline, and the proposed KG-5C-DETR, respectively. Compared with the baseline variants, KG-5C-DETR produces more accurate and spatially stable localization under weak-appearance conditions, further supporting the effectiveness of terrain-aware modulation when RGB cues are ambiguous.

### 4.5. Evolution of Detection Behavior Across Epochs

**Figure 8:**
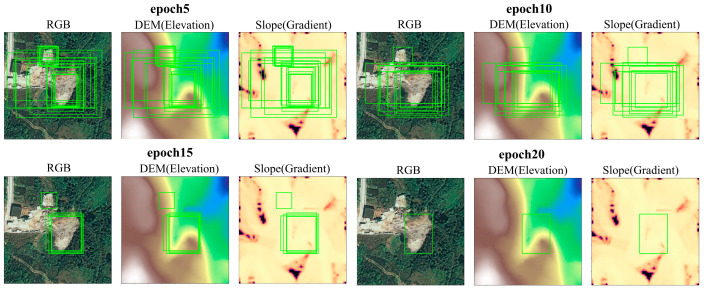
Evolution of predicted bounding boxes across training epochs on different modalities (RGB/DEM/Slope).

**Figure 9:**
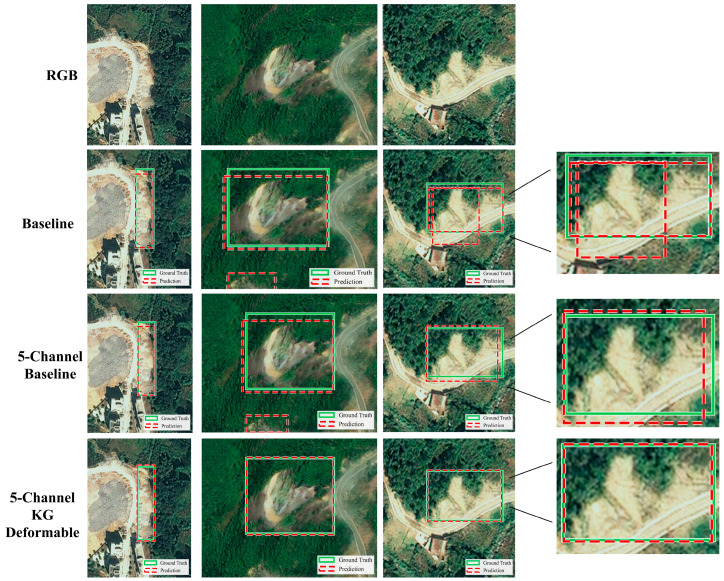
Visual comparison of detection results across model variants.

To visualize how predictions stabilize during training, Figure 8 tracks bounding-box refinement across early epochs (e.g., 5, 10, 15, and 20). At early stages, the detector produces multiple scattered candidate boxes, reflecting uncertainty under complex backgrounds. As training progresses, redundant proposals are suppressed and predictions converge to fewer, tighter boxes that better match the landslide extent. Compared with the baseline, KG-5C-DETR exhibits a more coherent refinement process, with fewer scattered proposals and more stable final localization. A direct qualitative comparison across model variants is further presented in Figure 9.

### 4.6. Failure Cases and Limitations

**Figure 10:**
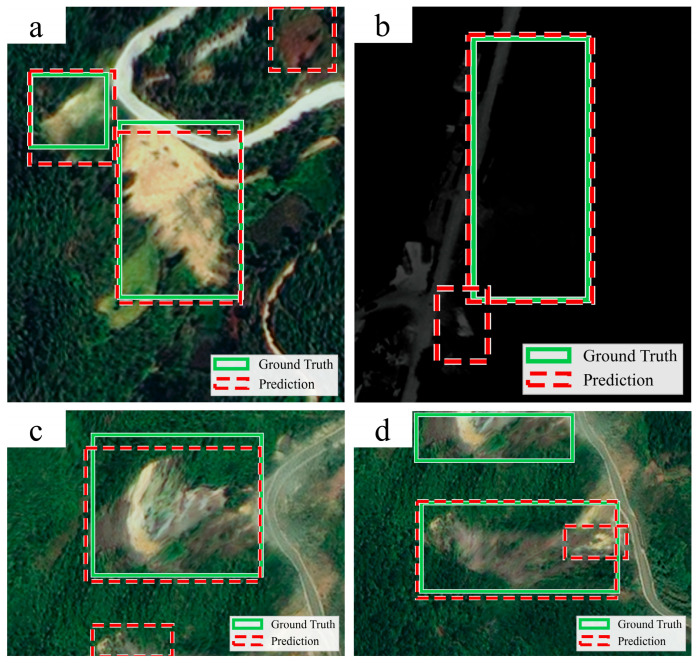
Representative failure cases of the proposed method. (**a**) False positives in road-adjacent and visually disturbed background; (**b**) false positives under low-contrast/dark-background conditions; (**c**) partial miss with additional false positives in weak-appearance scenes; (**d**) incomplete detection and background confusion in complex mountainous terrain.

Although the proposed method improves overall detection accuracy and localization robustness, several failure modes remain, as illustrated in Figure 10. First, in road-adjacent or visually disturbed areas, the model may produce false positives because non-landslide bare surfaces can resemble landslide scars. Second, under low-contrast and dark-background conditions, the detector may generate fragmented or spatially unstable predictions. Third, for elongated or weak-boundary landslides, only part of the target may be detected, or the landslide may be missed entirely. These cases indicate that DEM- and slope-derived priors are helpful but not sufficient to fully characterize landslide processes in all scenarios. Additional information such as lithology, vegetation indicators, hydrological conditions, or SAR/InSAR observations may further improve model robustness in future work.

### 4.7. Interpretability and Attention Analysis

**Figure 11:**
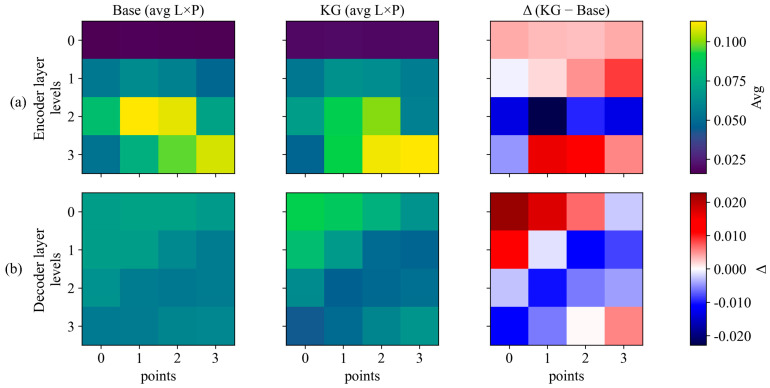
Statistical summary of deformable attention weights across feature levels and sampling points. (**a**) Encoder attention distribution;(**b**) Decoder attention distribution.

To further examine the effect of knowledge guidance, we analyzed the internal response patterns of the model. Figure 11 provides a statistical summary of deformable attention weight distribution across feature levels and sampling points. Within this figure, panel a and panel b display the summarized patterns for the encoder and decoder layers, respectively. The difference maps, which represent the subtraction of the baseline from the knowledge-guided results, indicate a redistribution of attention after terrain-aware modulation is introduced. This tendency suggests that the proposed module encourages the model to place greater emphasis on terrain-salient spatial patterns, although these attention maps should be interpreted as qualitative evidence rather than direct physical validation.

**Figure 12:**
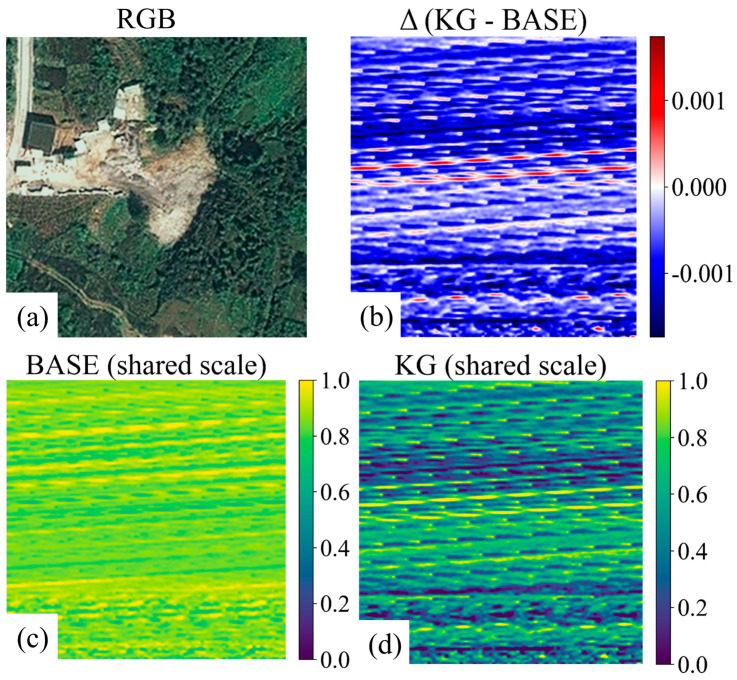
Unified-scale spatial response maps before and after knowledge guidance. (**a**) Baseline response map; (**b**) KG-5C-DETR response map; (**c**) Difference map (KG − Baseline); (**d**) Overlay visualization highlighting response changes.

This trend is further illustrated in Figure 12a–d using unified-scale spatial response maps and their comparative visualization. Figure 12a–d together suggest that terrain-aware modulation redistributes model responses toward topographically salient regions, such as scarp-like boundaries and locally high-gradient zones. Compared with the baseline responses, the knowledge-guided model exhibits fewer spurious activations in some relatively stable background areas. However, these visualizations provide qualitative interpretive support only and should not be regarded as direct evidence of physical landslide mechanisms.

## 5. Discussion

The primary challenge in landslide detection from satellite-scale (10 m) optical imagery resides in the spectral–textural ambiguity between landslide scars and non-landslide features—such as bare soil, active quarries, and road-cut slopes—which frequently co-occur in rugged mountainous environments. Our results demonstrate that conventional RGB-based detectors are prone to triggering spurious alarms in geomorphologically implausible locations [24,27,28].

### 5.1. Contribution of Terrain Cues Versus Knowledge-Guided Modulation

Our experiments effectively disentangle two distinct effects: the baseline benefit of multimodal input and the specific advantage of our integration mechanism. As summarized in Table 6, transitioning from RGB-only to a five-channel early-fusion baseline (M3) improves the AP@[0.5:0.95] from 67.8% to 71.5%. This indicates that DEM and slope provide essential context, aligning with geomorphological principles that identify terrain steepness as a fundamental control on slope stability [5,10,16,25,26].

Crucially, although the five-channel baseline and KG-5C-DETR utilize identical input data, our knowledge-guided modulation (M4) yields a further substantial gain (AP75: 76.2% → 77.2%) with negligible parameter overhead. This validates our hypothesis that naïve early fusion is insufficient [28,29,30]; the explicit injection of terrain priors into the feature hierarchy is necessary to fully exploit topographic information [36,37,38].

### 5.2. Why Knowledge-Guided Modulation Helps in Transformer-Based Detection

While transformer-based detectors are effective at modeling long-range context, their attention allocation may still be influenced by locally salient optical textures when spectral cues are ambiguous [27,31,34]. In KG-5C-DETR, terrain priors act as a learnable modulation signal that biases feature processing toward topographically plausible regions [39,40].

The interpretability analysis in Figure 11 and Figure 12 provides qualitative support for this behavior. Figure 11 summarizes deformable attention allocation across feature levels and sampling points and shows a redistribution under knowledge guidance (Δ = KG − Base) [41,42,43]. Figure 12 further illustrates this tendency using unified-scale spatial response maps and the corresponding difference map. Compared with the baseline responses, activations are relatively suppressed in stable, low-gradient background regions and enhanced around scarp-like boundaries and locally high-gradient zones [44,45,46]. This response pattern is consistent with the improved precision–recall behavior shown in Figure 6, especially under the stricter AP75 criterion.

Overall, these observations suggest that knowledge-guided modulation helps the detector place greater emphasis on terrain-salient regions when appearance cues alone are ambiguous. However, these visualizations should be interpreted as qualitative evidence only and not as direct proof of physical landslide mechanisms.

### 5.3. Limitations and Implications for Operational Screening

Despite the performance gains, certain challenges persist for potential operational screening. First, anthropogenic features (e.g., quarries) remain a primary source of residual false positives due to their landslide-like topographic signatures [17,20,33]. Second, the efficacy of the framework is intrinsically linked to DEM quality. Coarse or noisy elevation data may smooth subtle topographic markers, potentially leading to omissions of small-scale landslides [25,26].

Future research will focus on incorporating multi-source and multi-temporal cues, including LiDAR-assisted geometric extraction and Earth-observation deep learning strategies, to better distinguish natural failures from human activities [47,48,49]. Furthermore, we intend to investigate additional environmental predictors, such as the Topographic Wetness Index (TWI) and vegetation proxies (e.g., NDVI), to enhance robustness across diverse climatic regimes and human-modified landscapes [31,35].

In addition, the current experiments are conducted on a single benchmark dataset, and the generalizability of the framework across regions with different geomorphic and climatic conditions still requires further validation.

The results suggest that incorporating terrain priors plays a critical role in improving landslide detection performance. Compared with purely data-driven approaches, the proposed framework provides more robust feature representations under complex environmental conditions [27,28,29,30,31,48,49].

## 6. Conclusions

This study proposed KG-5C-DETR, a knowledge-guided five-channel Deformable Transformer framework for landslide detection that integrates optical imagery with terrain-derived priors. By injecting DEM- and slope-based knowledge into the multi-scale feature-learning process, the framework enhances the use of topographic context without increasing the input dimensionality.

Experimental results show that KG-5C-DETR consistently outperforms RGB-only and conventional early-fusion baselines in both detection accuracy and localization quality. In particular, the model achieves clear improvements under stricter localization criteria and exhibits stronger robustness in challenging scenarios such as vegetation interference, road-adjacent landslides, and dark-background targets.

Overall, this work demonstrates that incorporating geomorphological knowledge into transformer-based detection offers a more robust and interpretable strategy for automated landslide inventory mapping in complex mountainous environments. The proposed framework provides a practical basis for rapid post-event hazard assessment and may be extended in future studies through multi-temporal and multi-source information integration.

Nevertheless, several limitations should be noted. First, the proposed terrain prior is derived only from DEM and slope, and therefore does not explicitly encode lithological, structural, hydrological, or deformation-related information. Second, all experiments were conducted on the Bijie dataset, so the reported results mainly demonstrate within-dataset spatial generalization rather than universal transferability. Third, seasonal variation was not independently evaluated due to limited temporal stratification in the public dataset. Future work will incorporate richer geoscientific predictors, such as lithological maps, hydrological indices, vegetation indicators, and SAR/InSAR observations, and will further assess cross-region and cross-season generalization.

## Figures and Tables

**Table 1:** Multimodal data sources, preprocessing, and output format for landslide detection.

Input Channel	Source/Resolution	Preprocessing	Output
RGB (3-ch)	Sentinel-2 MSI, 10 m	Tiling 512 × 512; ImageNet mean/std normalization	3 × H × W
DEM (1-ch)	Per-tile raster (grayscale PNG)	Resized to match RGB; scaled to [0, 1]	1 × H × W
Slope (1-ch, °)	Per-tile raster (grayscale PNG)	Resized to match RGB; scaled to [0, 1]; unit: degrees (°)	1 × H × W

**Table 2:** Quantitative summary of the Bijie dataset.

Split	Image Tiles	Landslide Instances	Small	Medium	Large
Train	3514	4685	333	3116	1236
Val	197	251	17	142	92
Test	221	274	34	183	57

**Table 3:** Configuration of the knowledge-guided (KG) modulation module.

Component	Specification
Inputs	DEM (1) + Slope (1) from the same five-channel input
Normalization	Per-scene min–max normalization for DEM and slope
Terrain embedding network ϕ(·)	Two 3 × 3 conv layers + ReLU
Output channels of terrain prior (d_mod)	256
Per-level resize	Terrain prior tensor is resized to each feature level F_i_ spatial resolution
1 × 1 projection	Learnable 1 × 1 conv to match channels of each feature level
Activation	$\mathrm{Sigmoid}\;\mathrm{to}\;\mathrm{produce}\;\mathrm{modulation}\;\mathrm{mask}\;M_{i}\in \left[0,1\right]$
Modulation formula	$X_{l}^{\prime }=(1+\alpha M_{l})⊙X_{l}$
Modulation strength α	1.0
Insertion point	Before deformable attention encoder (multi-scale features modulated prior to transformer encoding)

**Table 4:** Model configurations for evaluating input modalities and the knowledge-guided (KG) module.

Model	Input Channels	Knowledge-Guided Module	Description
M1	RGB	×	Baseline Deformable DETR using optical imagery only, relying solely on spectral and textural cues
M2	RGB + DEM	×	Incorporates elevation information to introduce global topographic context and terrain structure
M3	RGB + DEM + Slope	×	Further integrates slope information to enhance sensitivity to terrain steepness and geomorphic discontinuities
M4 (KG-5C-DETR)	RGB + DEM + Slope	√	Proposed knowledge-guided five-channel Deformable DETR that injects DEM and slope-derived terrain priors into the detection framework

**Table 5:** Training and implementation settings for all experiments.

Item	Setting/Value
Framework	PyTorch v2.4.0+cu121
Hardware	1× NVIDIA RTX 4070 Ti (16 GB)
Backbone	ResNet-50 (ImageNet pretrained)
Transformer encoder/decoder	6 encoder layers/6 decoder layers
Hidden dimension	256
Feature levels	4
Attention heads	8
FFN dimension	1024
Dropout	0.1
Deformable attention sampling points	4
Object queries	300
Batch size	2
Training epochs	200
Optimizer	AdamW
Learning rate	2 × 10^−5^ (backbone lr 5 × 10^−6^)
Weight decay	1 × 10^−4^
AdamW betas	(0.9, 0.999)
LR schedule	Step decay at epoch 150 with factor γ = 0.1
Data augmentation	Random horizontal flip (*p* = 0.5); multi-scale resizing with aspect-ratio preservation
Loss function	$L=L_{cls}+\lambda_{1}L_{bbox}+\lambda_{2}L_{giou}$
Loss weights	$\lambda_{1}=5\;\;\;\lambda_{2}=2$
Input tile size	512 × 512
Random seed	42

**Table 6:** Quantitative comparison of Deformable DETR baselines and the proposed KG-5C-DETR on the held-out region-level test set.

Metric	YOLOv8n	YOLOv10n	M1 (RGB)	M2 (RGB + DEM)	M3 (RGB + DEM + Slope)	M4 (KG-5C-DETR)
Params (M)	39.85	39.86	39.85	39.86	39.86	39.87
AP@[0.5:0.95] (%)	50.9	63.4	67.8	69.5	71.5	72.9
AP50 (%)	77.3	81.2	91.8	93.5	93.8	94.4
AP75 (%)	63.7	67.5	72.0	75.0	76.2	77.2
APS (%)	40.6	43.4	35.0	39.7	47.1	48.8
APM (%)	53.7	58.4	68.8	70.0	72.5	73.1
APL (%)	60.4	64.2	71.6	72.8	73.0	74.1

## Data Availability

The Bijie Landslide Remote Sensing Dataset used in this study is publicly available on the official data repository of Wuhan University at: https://gpcv.whu.edu.cn/data/data.html (accessed on 21 April 2026). No new data were created or analyzed in this study.

## Abbreviations

The following abbreviations are used in this manuscript:

CNN	Convolutional Neural Network
DEM	Digital Elevation Model
DETR	DEtection TRansformer
KG	Knowledge-Guided
KG-5C-DETR	Knowledge-Guided Five-Channel Deformable DETR
mAP	Mean Average Precision
PR	Precision–Recall
RGB	Red–Green–Blue
RS	Remote Sensing
ViT	Vision Transformer
